# Immediate hypersensitivity to COVID-19 vaccines: Focus on biological diagnosis

**DOI:** 10.3389/falgy.2022.1007602

**Published:** 2022-09-30

**Authors:** Pascale Nicaise-Roland, Vanessa Granger, Angèle Soria, Annick Barbaud, Marc Pallardy, Sylvie Chollet-Martin, Luc de Chaisemartin

**Affiliations:** ^1^ Service d’Immunologie Biologique, Hôpital Bichat, DMU BIOGÉM, APHP, Paris, France; ^2^ Université Paris Cité, Inserm PHERE, Paris, France; ^3^ Université Paris-Saclay, Inserm, Inflammation Microbiome Immunosurveillance, Orsay, France; ^4^ Département de Dermatologie et Allergologie, Sorbonne Université, Hôpital Tenon, Paris, France; ^5^ Centre D'immunologie et des Maladies Infectieuses - Paris (Cimi-Paris), INSERM, Paris, France; ^6^ Département de Dermatologie et Allergologie, Sorbonne Université, INSERM, Institut Pierre Louis D'Epidémiologie et de Santé Publique, AP-HP. Sorbonne Université, Hôpital Tenon, Paris, France

**Keywords:** COVID-19 vaccine, anaphylaxis, basophil activation test, IgE, complement

## Abstract

Soon after the release of the new anti-COVID mRNA vaccines, reports came in from the US and the UK of anaphylactic reactions. Fueled by the necessary caution toward these new vaccine platforms, these reports had a great impact and were largely commented upon in the scientific literature and global media. The current estimated frequency is of 5 cases per million doses. Very little biological data are presented in the literature to support the anaphylaxis diagnosis in these patients in addition to skin tests. Allergic reactions to vaccines are rare and mostly due to vaccine excipient. Therefore, the poly-ethylene-glycol (PEG) present in both mRNA formulation, and already known to be immunogenic, was soon suspected to be the potential culprit. Several hypersensitivity mechanisms to PEG or to other vaccine components can be suspected, even if the classical IgE-dependent anaphylaxis seems to be one of the most plausible candidates. In the early 2022, the international guidelines recommended to perform skin prick tests and basophil activation tests (BAT) in people experiencing allergic reaction to the first dose of COVID-19 vaccine or with a history of PEG allergy. The aim of this review is to discuss the main potential mechanisms of immediate allergy to COVID19 vaccines based on published data, together with the various techniques used to confirm or not sensitization to one component.

## Introduction

In the context of COVID-19 pandemic, several vaccines have been developed in a few months, and the number of companies involved in vaccine development is increasing. These vaccines are presented in Table 1. Their effectiveness in reducing severe cases is remarkable. However, the existence of adverse events in particular potential allergic reactions has been rapidly reported. Indeed, severe immediate allergic reactions to the COVID-19 vaccines were described very early after the beginning of vaccination in the United States and the United Kingdom, and then all over the world. The more recent reports estimate that anaphylaxis cases for both Pfizer BNT162b2 and Moderna mRNA-1273 vaccines exhibit an estimated frequency of 11.1 to 12.4 and 2.5 to 20.4 cases per million doses administered, respectively (1, 2). Altogether, the number of doses given in the European Union as of June 2022 are the following : 649 million of Comirnaty, 155 millions of Spikevax, 69 millions of Vaxevria, 19 millions of Jcovden and 216,000 of Novavax. The existence of poorly understood severe reactions indirectly contributed to limiting vaccine access by fueling some reluctance to vaccination in the early 2021. To address this issue, a better knowledge of these reactions and of their mechanisms was urgently needed and led to several studies. Beside the identification of the mechanism(s) involved in allergic reactions, the identification of the culprit allergen(s) has also been evaluated.

**Table 1:** Composition of the vaccines approved by the European medical agency (**potential allergens in bold**).

* *	BNT162B2 Pfizer/BioNTech Cominarty	BNT162B2 bivalent Pfizer/BioNTech Cominarty Original/BA	mRNA-1,273 Moderna Spikevax	mRNA-1,273.214 Moderna	ChAdOx1-S AstraZeneca Vaxzevria	NVX-CoV2373 Novavax Nuvaxovid	Ad26.COV2-S Janssen-Cilag Jcovden	VLA2001 Valneva Valneva
Type of vaccine	mRNA coding for SARS-CoV2 spike glycoprotein	Bivalent vaccine: addition of mRNA coding for spike from BA1 omicron variant to the initial vaccine	mRNA coding for SARS-CoV2 spike glycoprotein	Bivalent vaccine: addition of mRNA coding for spike from BA1 omicron variant to the initial vaccine	Chimp adenovirus vector encoding SARS-CoV2 spike glycoprotein	Recombinant adjuvanted SARS-Cov2 spike protein	Adenovirus type 26 encoding SARS-CoV2 spike glycoprotein	Inactivated adjuvanted adsorbed SARS-Cov2 virus
Active substance **Potential allergens**	mRNA (30 *µ*g) **polyethylene glycol 2,000** **tromethamine and tromethamine hydrochloride (only in ready to use vials)**	mRNA (30 *µ*g booster dose)) **polyethylene glycol 2,000** **tromethamine and tromethamine hydrochloride**	mRNA (100 *µ*g) **polyethylene glycol 2,000** **tromethamine and tromethamine hydrochloride**	mRNA (50 *µ*g booster dose) **polyethylene glycol 2,000** **tromethamine and tromethamine hydrochloride**	recombinant ChAdOx1-S, produced by HEK 293 cells **polysorbate 80**	Recombinant adjuvanted spike protein produced in Spodoptera frugiperda Sf9 insect cells **polysorbate 80**	Recombinant Ad26. COV2-S produced in PER.C6 Tet R cells **Polysorbate 80**	Wuhan strain hCoV-19 produced on Vero cells, adsorbed on Aluminium hydroxide

The mechanisms of drug-induced anaphylaxis can be immunological, involving IgE-mediated basophil and mast cell activation, or IgG-mediated with activation of neutrophils and possibly monocytes and platelets; in other cases, it mainly relies on pharmacological activation of mast cells *via* complement activation or engagement of MRGPRX2 (3). All these pathways have been investigated in COVID-19 vaccine-induced anaphylaxis by preliminary studies, sometimes controversial, that will be discussed in the present review. These recent information on the potential immediate hypersensitivity mechanisms led to the establishment of clinical (skin testing) and biological guidelines to (1) evaluate the risk of a second vaccine dose and propose a safe alternative for at-risk patients, and (2) identify at-risk patients with an history of a previous allergic reaction to one of the vaccine components.

Beside these immediate hypersensitivity reactions, some delayed reactions have been reported in less than 0.3% which were mostly mild and did not contraindicate subsequent vaccinations (4). These reactions will not be discussed in this review.

## Potential mechanisms of COVID 19 vaccine-induced immediate hypersensitivity

**Figure 1:**
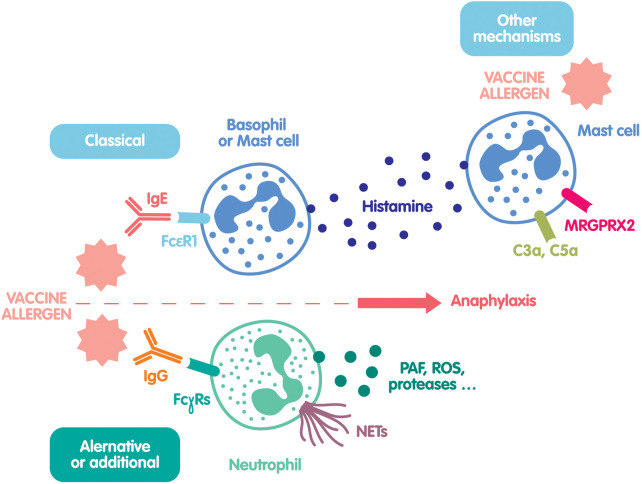
Legend. Main mechanisms of potential COVID-19 vaccine-induced hypersensitivity. The classical mechanism involves specific IgE-dependent mast cell and basophil activation leading to histamine/tryptase release. The alternative or additional mechanism involves specific IgG-dependent neutrophil activation leading to the release of reactive oxygen species (ROS), proteases such as elastase or neutrophil extracellular traps (NETs). Finally, several other mast cell activation mechanisms are suspected to play a role *via* C3a or C5a fixation to their receptors, or *via* the direct activation of MRGPRX2 by the vaccine.

The hypotheses regarding the mechanisms of anaphylactic reactions induced by mRNA vaccination against SARS-CoV-2 are multiple, and probably correspond, at least in part, to the classic mechanisms of drug anaphylaxis (5). Moreover, their rate is close the anaphylaxis rate to other vaccines (6). The first hypothesis is an IgE- or IgG-dependent mechanism linked to the presence of allergenic substance(s) in these vaccines which implies prior exposure and sensitization. However, the clinical reactions could also be linked to pseudo-allergic phenomena such as complement activation (complement activation-related pseudo-allergy or CARPA) without prior exposure, or the Mas-related G protein Receptor X2 receptor (MRGPRX2) engagement (7, 8) (Figure 1).

### IgE-mediated basophil and mast cell activation

IgE-mediated anaphylaxis implies a first exposure to an allergen leading to the production of specific IgE. These IgE bind to the high affinity receptors Fc*ε*RI on mast cells and basophils. Upon a new encounter, the allergen or a closely related substance activates mast cells and basophils by surface IgE cross-binding, which triggers degranulation of various mediators such as histamine or tryptase. This mechanism is the basis for routine anaphylaxis biological diagnosis, which encompass degranulated tryptase and histamine measurement, as well as specific IgE assessments. A true IgE-mediated allergic reaction to COVID-19 vaccines is possible, mainly based on documented PEG-mediated reactions in the literature, but seems very rare, as we'll see below.

### IgG-mediated anaphylaxis

Up to 30% of patients with clinically proven drug anaphylaxis do not have any sign of an IgE-dependent mechanism (9). Our group has demonstrated in various mice models that anaphylaxis can be triggered by a pathway involving specific IgGs that activate neutrophils (10, 11). Activated neutrophils release platelet-activating factor (PAF), a potent vasoactive lipid with effect similar to histamine. In a multicentric clinical study, we were able to confirm this mechanism in human, and showed that signs of neutrophil activation (in particular degranulation of neutrophil elastase and production of neutrophil extracellular traps) were correlated with severity in perioperative anaphylaxis patients (9).

### Complement activation and mast cell degranulation

Besides these two mechanisms, other pathways have been proposed to explain anaphylaxis that do not rely on the adaptive immune response. Since they do not require previous sensitization, these mechanisms may explain reactions observed to the first allergen exposure. Most of these mechanisms involve pharmacological activation of mast cells by the allergen. Some allergens have been described to activate the complement system, releasing C3a and C5a cleavage fragments that are able to trigger mast cell degranulation through specific receptors. These adverse effects known as CARPA have been documented with nanomedicines in experimental models but evidence in human are lacking (12). Moreover, Szebeni group also reported anti-PEG IgG-triggered complement terminal complex-mediated damage to PEGylated nanomedicines, that could decrease the efficacy of the nanomedicine and increase the toxicity *via* this complement activation (13).

### Mas-related G protein-coupled receptor X2 (MRGPRX2) engagement on mast cells

Mast cell direct activation by positively charged substances like iodinated contrast media, quinolones, or some neuromuscular blocking agents has been described through the Mas-Related G Protein coupled Receptor X2 (MRGPRX2) (14). Interestingly, mRNA stabilization with PEG induces also a positive charge that could make this mechanism possible during COVID19 vaccine reaction. Whether basophils can also express MRGPRX2 at their surface upon activation remains controversial, but would be of great interest in assessing COVID19 vaccine-related hypersensitivity (15). However, it was recently shown that tryptase release by activated mast cells cannot discriminate between IgE- and MRGPRX2-related mechanisms, leaving yet unanswered questions concerning this interesting receptor (16).

### Release of other active mediators

Finally, many mediators like prostaglandins, bradykinin, serotonin or nitric oxide could mimic anaphylaxis symptoms by inducing vasodilation or bronchoconstriction, and their potential contribution to anaphylaxis is only beginning to be investigated.

## Potential allergens in COVID 19 vaccines

Allergic reactions to vaccines are mostly due to excipients or contaminants, and exceptionally to the antigens themselves (3). The potential allergens contained in the vaccines that are available in the European Union are listed in Table 1.

Both mRNA vaccines (Cominarty and Spikevax) have a similar structure: they contain no protein or adjuvant, but only the mRNA which is packed with stabilizing lipids inside a lipidic nanoparticle covered with polyethylene glycol (PEG) to increase water solubility*.* While PEG has been the first suspected candidate, other components must be evaluated (17).

**PEG or macrogol** is an ether polymer with a molecular weight ranging from 200 to 35,000 g/mol. It is used in many industrial products, either pure in preparation for colonoscopy and laxatives, or as an excipient in some food, cosmetics, topical drugs, or therapeutic proteins. Anaphylaxis to PEG-containing products remains rare but have been reported (18). These reactions were mostly with high molecular weight PEG (>2,000 g/mol), both with oral route (19) or injected drugs (20)*.* Positive skin tests have been reported in PEG allergic patients, and specific IgG and IgE have been recently reported in some patients with severe reactions to injectable drugs and therapeutic protein (21, 22). This shows that PEG can be recognized by the immune system and can trigger the classical IgE pathway mechanism (23). The role of PEG IgG is less clear in this context. It has been suggested that specific IgG could activate the complement *via* the classical pathway, which in turn could activate mast cells *via* the anaphylatoxins. However, the prevalence of these IgG is high in patients exposed to PEG without any allergic reaction. Very recently, a time-course study of anti-PEG IgG did not evidence any increase in concentrations after each dose of mRNA vaccine, regardless of the vaccine used (24) A more detailed analysis of IgG subclasses involved, and the measurement of their affinity could help to distinguish harmful IgG susceptible of triggering a reaction. Moreover, it has been demonstrated that PEG itself can directly activate the complement system *via* the lectin and the alternative pathway (13, 25–27) and that lipid-conjugated PEG could be involved in the allergic reactions rather than PEG alone (28).

In addition to PEG, Moderna mRNA-1273 vaccine (Spikevax) also contains **tromethamine (or trometamol**), a widely used buffering agent. Some cases of anaphylaxis have been published to injectable drugs where tromethamine was identified as the culprit agent (29, 30). In the second version of Cominarty vaccine (ready to use vials), tromethamine has also been added. Very recently, bivalent mRNA vaccines from Pfizer (Cominarty Original/BA) and Moderna (mRNA-1273.214) have been approved by the EMA. mRNA coding for spike from BA1 omicron variant have been added to both original vaccines. However no other modification of the vaccine composition can be noticed, in particular concerning potential allergens.

A third vaccine, widely used in Europe, is a viral vector from a chimpanzee adenovirus coding for SARS-CoV2 spike protein (ChAdOx-1-S, AstraZeneca). It does not contain adjuvant either, but contains **polysorbate 80 (or Tween 80)**, a non-ionic detergent with poly(ethylene oxide) side chains that are similar to the PEG structure. Anaphylaxis to polysorbate 80 has also been observed, with cross-reactivity to PEG components (25, 31).

Two vaccines consisting in recombinant spike proteins are also available in the European Union : Nuvaxovid (recombinant adjuvanted spike protein produced in Spodoptera frugiperda Sf9 insect cells) and JCovden (Recombinant Ad26. COV2-S produced in PER.C6 Tet R cells). They both contain **polysorbate 80.**

Finally the Valneva vaccine, composed of inactivated adjuvanted adsorbed SARS-Cov2 virus does not contain any component suspected to induce allergic reaction.

In summary, most of COVID-19 vaccines contain a few potential allergens able to trigger anaphylaxis *via* several mechanisms incompletely understood (32). In addition to the clinical evaluation by allergologists and the use of skin tests in a stepwise fashion (33, 34), a biological evaluation can be done to get more information and determine the risk for vaccination or re-vaccination.

## Biological evaluation of COVID19 vaccine-induced allergy

### Anti-PEG antibodies

The few studies carried out on the presence of anti-PEG of the IgE isotype but also IgG and IgM have been done using “in-house” techniques (21) A recent commercial ELISA was studied in 20 patients known to have experienced clinical reactions to drugs containing PEG; in this work, 4 out of these 20 patients had anti-PEG 2,000 IgE, and all had positive PEG skin tests (35). On a technical level, it is important to note the possible interference of bovine serum albumin and Tween 20, often used in ELISA; skimmed milk and an alternative detergent would probably be more appropriate reagents (35). Flow cytometric methods have also been described to assay anti-PEG IgE (36). Interestingly, Zhou *et al.* (21) found anti-PEG IgE and IgG in patients who had an anaphylactic reaction to products for colonoscopy preparation containing PEG 3350. It seems that some of these antibodies preexist in the general population, with a frequency of anti-PEG IgG of 5 to 9%, which could explain the manifestations observed at first administration (37).

The recent results on the frequency of anti-PEG antibodies during post-vaccination reactions are contradictory. This may be partly due to a lack of standardization of assay methods and of the gradation of the severity of allergy to PEG (38, 39). Some authors detected neither anti-PEG IgE nor IgG in post-vaccination reactions (34, 40), others found IgE and IgM but their control population was small. One of the questions is whether it would not be preferable to develop techniques to search for antibodies directed against PEG in the form of nanoparticles, or even against the vaccine itself (41). New robust tests are needed.

### Proteins from complement activation

When hypothesizing CARPA-type mechanism, different complement activation parameters can be measured at the time of the reaction: anaphylatoxins C3a and C5a and the soluble fraction of the membrane attack complex C5b-9. In a pig experimental work, increased soluble C5b-9 levels correlated with the presence of anti-PEG IgM, after stimulation with PEGylated liposomes (32). Lim *et al.* (42) found increased C3a levels just after the clinical reaction in 3 patients, persisting from 48 h to one month. However, this increase was not confirmed by our group in 5 patients sampled at the time of the reaction (43). These preliminary results do not make it possible to conclude on the interest of these markers. Moreover, it is difficult to obtain a blood sample at the time of the clinical reaction, particularly in patients vaccinated outside a hospital.

### Mast cell activation and -derived mediators

To assess a possible mast cell degranulation in favor of an anaphylactic reaction induced by mRNA vaccines, histamine and tryptase assays could be informative. Very few studies report the measurement of tryptase at the time of the reaction, and they do not show any increased levels (26, 38, 42, 44, 45). Warren et al. study is the only one reporting elevated tryptase levels (between 14 and 25 *μ*g/l for a basal tryptase between 2 and 6 *μ*g/L) in 8 patients at time of the reaction (34). Our group reported increased histamine levels in 1 patient out of 5, within 30 min of the post-vaccination reaction, while tryptase levels were not modified (43).

Basal tryptase levels could also be of interest, even if no increased risk for reaction has been described in patients with mastocytosis (3). A few studies have shown a subnormal concentration in some patients: median of 8.5 to 12.8 *μ*g/l, i.e. above the 95th percentiles described in the general population (46, 47)**.** This could be in favor of gene duplication-related hyper-alpha − tryptasemia that needs to be better documented in the future (48). Moreover, the KIT D816V mutation research in the blood can be done to document mastocytosis, even in the presence of normal baseline tryptase (49).

### The basophil activation test

The basophil activation test (BAT) using CD63 and/or CD203 as activation markers by flow cytometry was developed as early as January 2021 to explore immediate hypersensitivity to mRNA vaccines. Various authors tried to determine its place in the management of patients who reported reactions to drugs containing PEG before the first dose (50), or experienced reactions just after the first dose. In both cases there was an urgent need to secure vaccine injections (46).

Most of the published studies have been done on small patient series. Troelnikov *et al*. (50) performed BAT with PEG 2,000 nanoparticles in 3 patients known for PEG allergy and evidenced basophil activation. Labella *et al.* (46) found a positive BAT to PEG 2,000 and to the vaccine in 5/16 patients. Warren *et al.* (34) reported a positive BAT in 10/11 patients tested in the presence of PEG 2,000 DMG in the form of nanoparticles and vaccine. The frequency of patients with positive BAT is therefore very variable and could depend on the patients (already known to react to PEG or not for example) and the stimuli used *ex vivo*, whole vaccine and PEG nanoparticles seeming to give the highest positivity. Different allergens can be used in BAT. PEG 2,000 and PEG 2,000 DMG have been recently marketed for this test. However, as PEG contained in the vaccines is in the form of nanoparticles conjugated with lipids, some authors carried out BAT in the presence of the vaccine and/or PEG in the form of lipid nanoparticles approaching the truly potential immunogenic form (34, 50). However, in the early 2022, some authors evidenced that BAT was positive in response to vaccine alone in 50% of the patients who had COVID, and did not react during the vaccine injection (46). This information, that remains to be confirmed, must encourage to interpret BAT results with caution, in particular in patients who experienced SARS-Cov 2 infection. However, most authors agree in concluding that in the event of an anaphylactic reaction after injection of an mRNA vaccine, BAT is more frequently positive than skin tests confirming an activation mechanism which would not necessarily be IgE dependent (34, 40, 50). In our group in Paris, preliminary data in 30 patients with anaphylaxis after the first injection of a mRNA vaccine confirm that BAT can be positive while skin tests are negative (Nicaise-Roland *P*, Soria A *et al.*, unpublished results). A recent Review by Eberlein *et al*. concluded that BAT helps elucidate allergic reactions to COVID-19 vaccines, but defining exact threshold of positivity is still needed (51).

We can thus assume that BAT is a quite simple and well-known test that needs to be further evaluated in larger well-characterized patients, with appropriate and standardized stimuli.

### The histamine release test

This test is only documented in two studies in this setting. The first one evidenced transient positive results in 3 patients who experienced a reaction (52), and the other one described positive results in 2/10 patients with positive skin tests to PEG (53).

## Conclusion

Eighteen months after the first vaccinations against COVID-19, the present real-world cohort survey can suggest that serious adverse effects are extremely rare. For instance, an analysis of 20,000 participants revealed that the adverse effects observed in 0.3% of the subjects were associated with full vaccination dose, vaccine brand, young age and COVID-19 (54). Research improved our understanding of COVID-19 vaccine allergy mechanisms, and made available some biological tools to an adequate management of the suspected patients (55, 56). Some tests, such as BAT, are now available to help the diagnosis in addition to skin tests. We can assume that BAT is the best biological tool to evaluate the *ex vivo* reaction to both whole vaccine and each excipient. The identification of the culprit agent even led to a safe and successful desensitization in a recent series of 6 patients (57). Conversely, the quantification of anti-PEG IgE or IgE cannot be recommended so far. Finally, lessons learned from nanomedicines need to be applied (58). There is a need to safely immunize patients who are at risk or who experienced immediate vaccine reactions, using antihistamines for example. Several studies are still ongoing in order to increase our knowledge and make large-scale vaccination safe and successful.
